# Early passaging of mesenchymal stem cells does not instigate significant modifications in their immunological behavior

**DOI:** 10.1186/s13287-018-0867-4

**Published:** 2018-05-02

**Authors:** Niketa Sareen, Glen Lester Sequiera, Rakesh Chaudhary, Ejlal Abu-El-Rub, Subir Roy Chowdhury, Vikram Sharma, Arun Surendran, Meenal Moudgil, Paul Fernyhough, Amir Ravandi, Sanjiv Dhingra

**Affiliations:** 1 Institute of Cardiovascular Sciences, St. Boniface Hospital Research Centre, Department of Physiology and Pathophysiology, University of Manitoba, Winnipeg, Canada; 2 Division of Neurodegenerative Disorders, St. Boniface Hospital Research Centre, Department of Pharmacology & Therapeutics, University of Manitoba, Winnipeg, Canada; 3 School of Biomedical and Healthcare Sciences, Plymouth University Peninsula Schools of Medicine and Dentistry, Plymouth, England

**Keywords:** Mesenchymal stem cells, Lipidomics, Proteomics, Immunoprivilege, Bioenergetics, Passage, Apoptosis

## Abstract

**Background:**

Bone marrow-derived allogeneic mesenchymal stem cells (MSCs) from young healthy donors are immunoprivileged and their clinical application for regenerative medicine is under evaluation. However, data from preclinical and initial clinical trials indicate that allogeneic MSCs after transplantation provoke a host immune response and are rejected. In the current study, we evaluated the effect of an increase in passage number in cell culture on immunoprivilege of the MSCs. Since only limited numbers of MSCs can be sourced at a time from a donor, it is imperative to expand them in culture to meet the necessary numbers required for cell therapy. Presently, the most commonly used passages for transplantation include passages (P)3–7. Therefore, in this study we included clinically relevant passages, i.e., P3, P5, and P7, for evaluation.

**Methods:**

The immunoprivilege of MSCs was assessed with the mixed leukocyte reaction assay, where rat MSCs were cocultured with peripheral blood leukocytes for 72 h. Leukocyte-mediated cytotoxicity, apoptosis (Bax/Bcl-xl ratio), leukocyte proliferation, and alterations in cellular bioenergetics in MSCs were assessed after the coculture. Furthermore, the expression of various oxidized phospholipids (oxidized phosphatidylcholine (ox-PC)) was analyzed in MSCs using a lipidomic platform. To determine if the ox-PCs were acting in tandem with downstream intracellular protein alterations, we performed proteome analysis using a liquid chromatography/mass spectrometry (LC/MS) proteomic platform.

**Results:**

Our data demonstrate that MSCs were immunoprivileged at all three passages since coculture with leukocytes did not affect the survival of MSCs at P3, P5, and P7. We also found that, with an increase in the passage number of MSCs, leukocytes did not cause any significant effect on cellular bioenergetics (basal respiration rate, spare respiratory capacity, maximal respiration, and coupling efficiency). Interestingly, in our omics data, we detected alterations in some of the ox-PCs and proteins in MSCs at different passages; however, these changes were not significant enough to affect their immunoprivilege.

**Conclusions:**

The outcome of this study demonstrates that an increase in passage number (from P3 to P7) in the cell culture does not have any significant effect on the immunoprivilege of MSCs.

**Electronic supplementary material:**

The online version of this article (10.1186/s13287-018-0867-4) contains supplementary material, which is available to authorized users.

## Background

Bone marrow (BM)-derived mesenchymal stem cells (MSCs) are attractive candidates for cell therapy since these cells are immunoprivileged [1]. Therefore, allogeneic (unrelated donor) MSCs can suppress the host immune system and survive after transplantation. In fact, the outcome of numerous preclinical studies and initial clinical trials have demonstrated that allogeneic BM-MSCs after transplantation were able to initiate a regenerative process [2]. However, long-term survival of transplanted MSCs was not detected in the recipient system. We recently reported in a rat model of myocardial infarction that allogeneic MSCs after transplantation into the infarcted heart lost their immunoprivilege and were rejected by the host immune system [3]. The outcome of our studies was confirmed by several other reports that allogeneic MSCs after transplantation become immunogenic and are rejected by the recipient immune system [4]. Therefore, for a successful bench-to-bedside translation of MSC-based regenerative therapies, it is imperative to understand the mechanisms of immune switch in MSCs from the immunoprivileged to immunogenic state.

To maintain uniformity in the quality of the cell product for any allogeneic cell-based clinical trial, MSCs derived from a single healthy donor are used for transplantation in multiple recipients. Therefore, it is imperative to expand them in culture to facilitate generation of the required number of MSCs. The safety of various passages of MSCs in the in-vitro studies has been amply demonstrated. A recent study reported no changes in telomeric ends for up to 25 passages [5]. However, subjecting MSCs to passages in cell culture, even in the short term, has been associated with physiological changes. Madeira et al. reported major differences in the pathways pertaining to culture-induced senescence [6]. There are numerous factors that may influence the overall optimum physiology of the cells, and these may be affected by the passaging of MSCs. The cell surface lipidome analysis of MSCs is garnering attention of late. The passaging of MSCs was found to alter levels of cell surface lipids and immune-modulation [7]. The oxidized phospholipids (oxidized phosphatidylcholine (ox-PC)), in particular, are known to affect the immunological behavior of cells [8]. Furthermore, cellular bioenergetics including cellular respiration and metabolic pathways are instrumental to stem cell renewal, maintenance, and general cell health [9]. Altered metabolic pathways lead to a difference in the functional capabilities of stem cells [10]. It is reported that, with an increase in passage number in the culture, cellular bioenergetics and growth rate are affected [11, 12].

Therefore, in this study, we have attempted to investigate the effect of an increase in passage number in cell culture on the immunological behavior of MSCs. The most commonly used passages for cell therapy in ongoing clinical studies include passage (P)3–7. In this study, we included the clinically relevant passages, i.e., P3, P5, and P7, for evaluation. We have employed functional evaluation as well as whole cell high-throughput assessment. We have chosen parameters including cell surface ox-PCs, cellular bioenergetics, global proteomic assessment, and general immune as well as cell survival pathways for our investigations. These characteristics have hitherto not been studied in detail. With MSCs poised at the cusp of clinical application, it is becoming increasingly apparent that our knowledge is very limited in various attempts to transfer understanding from the in-vitro setting into in-vivo applications.

## Methods

### Experimental animals

Unrelated male Sprague-Dawley (SD) rats (200–250 g) were used for the isolation of bone marrow MSCs and for the isolation of peripheral blood leukocytes. The study protocol was approved by the Animal Care Committee of the University of Manitoba and conformed to the ‘Guide for the Care and Use of Laboratory Animals’ published by the US National Institutes of Health (NIH Publication No. 85–23, revised 1985).

### MSC isolation and characterization

Bone marrow cells were flushed from the cavities of femur and tibias of SD rats. After the connective tissue was removed from around the bones, both ends were cut. The bone marrow plugs were flushed with Dulbecco’s modified Eagle’s medium supplemented with 15% fetal bovine serum (FBS), 100 units/ml penicillin G, and 0.1 mg/ml streptomycin. Cells were plated and cultured in the same medium followed by a media change and the removal of nonadherent hematopoietic cells the next day. The medium was replaced every 3 days, and the cells were subcultured when confluency exceeded 90%. MSCs from passages 3, 5, and 7 were used for the studies described herein.

MSCs were characterized by flow cytometry as described previously [3]; the cell population which was identified as CD90.1^+^, CD29^+^, CD45^−^, and CD34^−^ was used for further experiments. To further characterize the cells, MSCs were analyzed for their ability to differentiate into osteogenic, adipogenic, and chondrogenic lineages using a kit (R&D systems, catalogue number SC020). The cells were induced to differentiate and stained using reagents and primary antibodies provided in the kit (osteocalcin for osteogenic differentiation; FABP4 for adipogenic differentiation; aggrecan for chondrogenic differentiation). The secondary antibodies used (AF488 for osteocytes; AF647 for adipocytes and chondrocytes) were purchased separately. The nuclei were stained with DAPI. The images were captured using a Cytation5 at 20× magnification. Additionally, differentiated MSCS were also stained using Alizarin Red (osteogenic) and Oil Red-O stain (adipogenic).

### MSC population doubling

The population doubling time of MSCs at different passages was calculated using a trypan blue cell viability assay. The cells were plated at 100,000 cells/well in six-well dishes. After 96 h of culture, the MSCs were detached using trypsin EDTA followed by staining with trypan blue and counting of the live cell number using an automated cell counter (BioRad). The doubling time was calculated as follows:

$$ \mathrm{Doubling}\ \mathrm{time}=\mathrm{time}\ \mathrm{of}\ \mathrm{culture}\times \log (2)/\log\ \left(\mathrm{final}\ \mathrm{cell}\ \mathrm{number}\right)\hbox{--} \log\ \left(\mathrm{initial}\ \mathrm{cell}\ \mathrm{number}\right) $$

### Mixed leukocyte-mediated cytotoxicity

Leukocytes were isolated from rat spleen using HISTOPAQUE 1083 (Sigma-Aldrich) and cocultured with allogeneic MSCs at different passages (3, 5, and 7) at a ratio of 10:1 (leukocytes:MSCs). After 72 h of coculture, leukocyte-mediated cytotoxicity in MSCs was assessed by a Live/Dead viability/cytotoxicity assay kit (Thermo Fisher Scientific, L3224).

### Assessment of apoptosis

Apoptosis in MSCs at different passages (P3, P5, and P7) was assessed after 72 h of coculture with mixed leukocytes by measuring Bax and Bcl-xl levels using Western blot. Briefly, total protein levels were measured by Bradford protein assay, and 25 μg of protein was used in each group for SDS-PAGE electrophoresis. After separation with electrophoresis, proteins were transferred to PVDF membranes and probed with primary antibodies for Bax and Bcl-xl (Santa Cruz Biotechnologies Inc., CA, USA) and secondary antibodies (Biorad Inc.). The membranes were developed using x-ray film, and bands were quantified using Quantity One software for densitometry.

### Leukocyte proliferation

The effect of MSCs on the proliferation of leukocytes was analyzed using an MLR assay. Leukocytes were cocultured with allogeneic MSCs at different passages (P3, P5, and P7) for 72 h at a ratio of 1:10 (MSCs:leukocytes). The leukocyte proliferation was measured by flow cytometry (BD Accuri). Briefly, after coculture, the leukocytes in the supernatant were collected and spun at 1000 rpm for 5 min. The pellet was washed three times using 1× phosphate-buffered saline (PBS), and suspended in 100 μl cold PBS. The cells were then fixed using 5 ml ice-cold 70% ethanol followed by RNase (20 μg/ml) treatment for 30 min. The cells were then stained with propidium iodide (PI; 5 μg/ml) for 5 min at room temperature and analyzed using flow cytometry. To measure leukocyte proliferation, a cell cycle analysis was performed by counting the number of cells entering the S phase (proliferating phase) and the G2/M phase from the G0/G1 phase (resting cells) of the cell cycle.

### Assessment of the secretion profile of leukocytes

The leukocytes were cocultured with MSCs at different passages for 72 h at a ratio of 1:10 (MSCs:leukocytes). The cytokine secretion profile of leukocytes was analyzed using a multianalyte rat cytokine ELISArray kit (Qiagen; MER 336161) following instructions from the manufacturer. We analyzed the levels of 12 different cytokines including interleukin (IL)-1α, IL-1β, IL-2, IL-4, IL-6, IL-10, IL-12, IL-13, interferon (IFN)-δ, tumor necrosis factor (TNF)-α, granulocyte-macrophage colony-stimulating factor (GM-CSF), and RANTES. The plate was read at 450 and 570 nm using a Cytation5 analyzer (BioTek Inc.) in plate reader mode.

### Measurement of cellular bioenergetics

The cellular bioenergetics were determined using the extracellular flux (XF24) analyzer (Seahorse Bioscience). MSCs (4 × 10^4^ cells/well) and leukocytes were cocultured at a ratio of 1:10 (MSCs:leukocytes) in XF24 plates for 72 h. The mean basal respiration was determined by recording oxygen consumption rate (OCR) measurements before adding inhibitors or activators. ATP-linked OCR and proton leak were determined by injecting oligomycin (1 μM). The maximal respiration rate was determined after adding FCCP (an uncoupler of the electron transport chain) at a concentration of 1 μM. The difference between the basal rate and this FCCP-stimulated rate is the reserve capacity of the mitochondria, which is a measure of the maximal potential respiratory capacity the cell can utilize under conditions of stress and/or increased energetic demands. To completely inhibit mitochondrial electron transport, antimycin A (1 μM) and rotenone (1 μM) were used. The OCR determined after rotenone and antimycin A injection is attributable to nonmitochondrial oxygen consumption. Mitochondrial basal respiration, proton leak, and the maximal respiration were calculated after corrections were performed for the nonmitochondrial OCR for each assay. Under these conditions, viability was over 90% for all cell types and remained so over the time course of the assay. At the end of the assay period, trypsinized cells were collected, and values were normalized to the total cell number in each well [13].

### Oxylipidomic analysis

Cell surface oxidized phosphatidylcholine (ox-PCs) levels were measured in MSCs at different passages (P3, P5, and P7) by liquid chromatography/mass spectrometry (LC/MS) analysis. Total cellular lipids were extracted from cell pellets using a protocol adapted from Folch et al. [14]. Oxylipidomic analysis was performed with reverse-phase high-performance liquid chromatography (HPLC) using an Ascentis Express C18 column (Supelco Analytical, Bellefonte, PA, USA). Data were collected using analyst 1.6 software (Applied Biosystems, Canada) and quantified using MultiQuant 2.1 (Absciex, Ontario, Canada). The mass spectrometry data were log transformed and autoscaled (mean-centered and divided by standard deviation of each variable) before applying statistical analysis. To determine the changes in ox-PCs that were statistically significant between different passages P3, P5, and P7, we performed a one-way analysis of variance (ANOVA) with a *p* value cut-off of 0.05, followed by Tukey’s Honestly Significant Difference (Tukey’s HSD).

### E06 antibody treatment

E06 is a blocking antibody that specifically inhibits oxidized phosphatidylcholines in cells. To assess the involvement of ox-PCs in regulating leukocyte-mediated cytotoxicity, apoptosis, and cellular bioenergetics in MSCs, we cocultured MSCs and leukocytes with or without E06 antibody (1 ng/ml) (MSC + L + Ab) for 72 h and measured the abovementioned parameters.

### Proteomic analysis

#### Sample preparation for mass spectrometry

Whole cell proteomic analysis was performed in MSCs at different passages (P3, P5, and P7) by the LC/MS proteomic platform. MSCs were cultured at different passages, and cell pellets were collected and washed in ice-cold PBS (pH 7.2) followed by treatment with urea lysis buffer (8 M urea in 0.1 M Tris-HCl, pH 8.5). Protein estimation was performed by Qubit fluorescence assay (Invitrogen). A total of 50 μg protein was digested using the FASP procedure as described previously [15]. Liquid chromatography tandem mass spectrometric analysis of tryptic peptides (500 ng) was carried out using a Proxeon nano spray ESI source (Thermo Fisher, Hemel, UK) and analyzed using Orbitrap Velos Pro FTMS (Thermo Finnigan, Bremen, Germany) [16].

#### Proteomic data analysis by MaxQuant

Peptides and proteins were identified by Andromeda via an automated database search of all tandem mass spectra against a curated target/decoy database (using forward and reverse versions of the *Rattus norvegicus* [Taxonomy ID 10116]) and Uniprot protein sequence database (http://www.uniprot.org; release October 2015) containing all rat protein entries from Swiss-Prot and TrEMBL. Cysteine carbamidomethylation was searched as a fixed modification, whereas N-acetyl protein, deamidated NQ, and oxidized methionine were searched as a variable modification. The resulting Andromeda peak list-output files were further processed using MaxQuant software. The downstream bioinformatics data analysis was carried out using the Perseus software suite (1.5.0.15) and the Ingenuity Pathway Analysis software tool (Ingenuity Systems, Qiagen, Redwood City).

### Statistical analysis

Experimental values are expressed as mean ± SD. The comparison of mean values between various groups was performed by one-way ANOVA followed by multiple comparisons by Tukey test using the software GraphPad Prism. A *p* value < 0.05 was considered to be significant.

## Results

### Differentiation of MSCs

To characterize MSCs, cells were induced to differentiate toward the adipogenic, osteogenic, and chondrogenic lineages. Our data demonstrate that MSCs have the ability to differentiate toward these three lineages (Additional file 1: Figure S1).

### Population doubling of MSCs at different passages

**Fig. 1:**
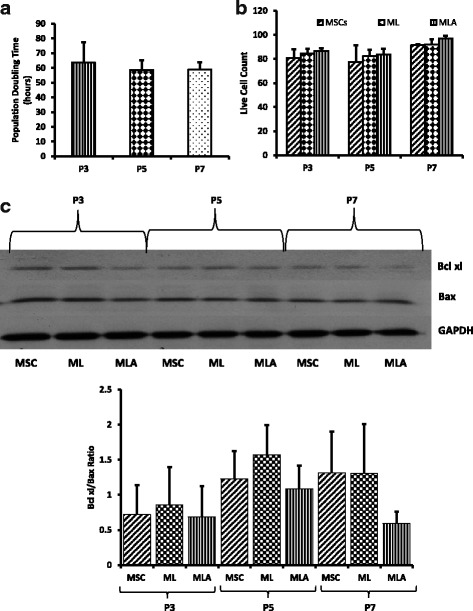
Assessment of doubling time and immunoprivilege of MSCs. **a** Population doubling of MSCs at different passages was determined using trypan blue cell viability assay. The cells were plated in equal numbers followed by calculating the live cell number after 96 h of culture. There was no significant difference found in population doubling time of cells at different passages. **b**, **c** MSCs were cocultured with leukocytes (with or without E06 blocking antibody) for 72 h at a ratio of 1:10 (MSCs:leukocytes). **b** Leukocyte-mediated cytotoxicity in MSCs at different passages was determined by cytotoxicity assay kit using flow cytometry. There was no significant difference found in the level of cytotoxicity at different passages in the presence of leukocytes alone or in the presence of leukocytes and E06 antibody. **c** Western blot analysis was performed to determine the levels of the pro- and antiapoptotic proteins Bax and Bcl-xL. There was no significant difference observed in the Bax/Bcl-xl ratio in MSCs at different passages in the presence of leukocytes alone or in the presence of leukocytes and E06 antibody. Data are represented as mean ± SD (*n* = 3–6). ML, MSCs + leukocytes; MLA, MSCs + leukocytes + E06 antibody; MSC, mesenchymal stem cells

To investigate the effect of an increase in passage number on population doubling time of MSCs, a cell viability assay was performed; our data demonstrate that there was no significant difference in the population doubling time of MSCs in the culture at P3, 5, or 7 (Fig. 1a).

### MSCs show no significant changes in immunological behavior with an increase in passage number

Bone marrow-derived MSCs are reported to be immunoprivileged and thus are able to escape the host immune system after transplantation. To understand the effect of an increase in passage number on the immunoprivilege of MSCs, cells at P3, P5, and P7 were cocultured with mixed leukocytes. The leukocyte-mediated cytotoxicity was measured in MSCs. Our results demonstrate that MSCs were immunoprivileged at all three passages (P3, P5, and P7) since we found more than 80% live cells even after 72 h of coculture with leukocytes (Fig. 1b). Interestingly, there was no significant difference detected in the number of live/dead MSCs at different passages after the coculture (Fig. 1b). We also assessed leukocyte-mediated apoptosis of MSCs and found no significant difference in the ratio of the antiapoptotic protein Bcl-xl and the proapoptotic protein Bax at P3, P5, and P7 after coculture with leukocytes (Fig. 1c).

**Fig. 2:**
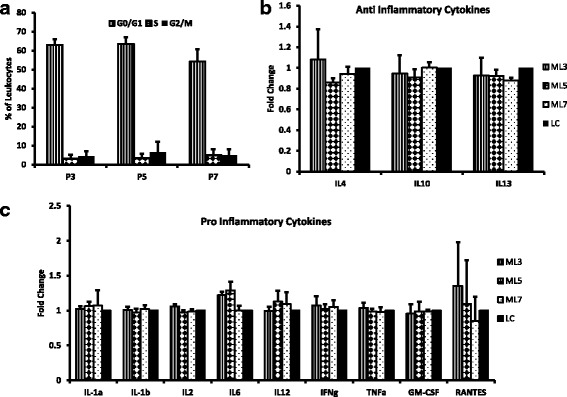
Effect of MSCs on leukocyte proliferation and the secretion profile of leukocytes. Leukocytes were cocultured with MSCs at passages 3, 5, and 7 at a ratio of 1:10 (MSCs:leukocytes) for 72 h. **a** Leukocyte proliferation was measured by flow cytometry. The data are represented as different stages of the cell cycle: G0/G1 phase, S phase, and M phase. The extent of leukocyte proliferation by MSCs did not change with an increase in passage number since there was no significant difference found in the number of leukocytes at different stages of the cell cycle among different passages. The effect of an increase in passage number of MSCs on the secretion of **b** anti-inflammatory and **c** proinflammatory cytokines by leukocytes was analyzed using ELISA array. The results indicate that MSCs at different passages had no significant effect on the secretion profile of leukocytes. Data are represented as mean ± SD (*n* = 3–4). GM-CSF, granulocyte-macrophage colony-stimulating factor; IFN, interferon; IL, interleukin; LC, leukocytes alone; ML3, leukocytes cocultured with MSCs at P3; ML5, leukocytes cocultured with MSCs at P5; ML7, leukocytes cocultured with MSCs at P7; TNF, tumor necrosis factor

Mesenchymal stem cells have the ability to suppress immune cell proliferation and promote immune tolerance. We analyzed the effect of an increase in passage number of MSCs on their ability to suppress leukocyte proliferation and found that there were no significant differences in the level of suppression of leukocyte proliferation by MSCs at different passages (Fig. 2a). The data are represented as different stages of the cell cycle including G0/G1 phase, S phase, and G2/M phase. G1/G0 phase represents the stage where the cells prepare for the next division cycle by synthesizing proteins and RNA required for the division and multiplication. In S phase, the DNA synthesis occurs allowing the cells in G2/M phase to have double DNA which becomes divided equally in the cells once they undergo mitotic cell division. There were no significant differences observed at any stage among the different passages, indicating that the MSCs at P3, 5, and 7 affect leukocyte proliferation at the same rate.

To further assess the effect of MSCs at different passages on the immunomodulatory effects of leukocytes, we analyzed the levels of several proinflammatory cytokines including IL-1α, IL-1β, IL-2, IL-6, IL-12, IFN-γ, TNF-α, GM-CSF, and RANTES, and the anti-inflammatory cytokines IL-4, IL-10, and IL-13 in leukocytes after coculture with MSCs. Our data demonstrate that there was no significant change in the levels of these soluble factors in leukocytes after coculture with MSCs at different passages (Fig. 2b, c). These results suggest that an increase in the passage number from P3 to P7 does not affect immunoprivilege and immune tolerance of MSCs.

### Effect of an increase in passage number on cellular bioenergetics

**Fig. 3:**
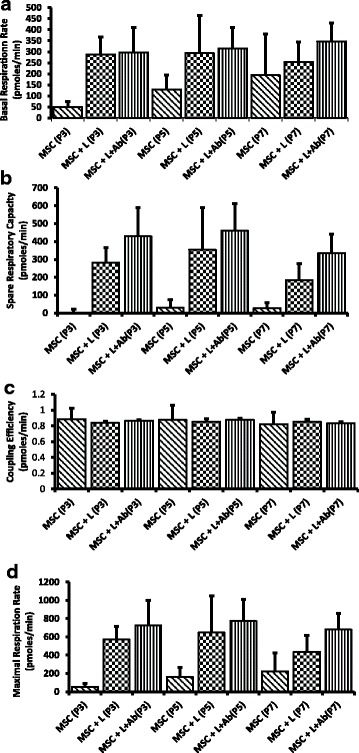
Measurement of cellular bioenergetics using XF24 Seahorse analyzer. Mesenchymal stem cells (MSCs) at passages 3, 5, and 7 were cocultured with leukocytes (with or without E06 blocking antibody) at a ratio of 1:10 (MSCs:leukocytes) for 72 h. **a** Basal respiration rate, **b** spare respiratory capacity, **c** coupling efficiency, and **d** maximal respiration rate were measured in MSCs. There were no significant changes observed in these parameters in MSCs with the increase in passage number, in the presence of leukocytes, and in the presence of E06 antibody. The values are normalized to cell number in each well. Data are represented as mean ± SD (*n* = 4–6). MSC + L,  MSCs + leukocytes; MSC + L + Ab,  MSCs + leukocytes + E06 antibody

It is reported that intracellular energy metabolism has a primary influence on the presence or absence of T-cell activation signals. Therefore, cellular bioenergetics are a key factor for determining the response of transplanted cells toward the host immune system. We assessed the effect of an increase in passage number on the intracellular energy metabolism using a SeaHorse Bioscience XF24 analyzer. We found no significant difference in basal respiration rate and spare respiratory capacity in MSCs at P3, P5, and P7 before and after coculture with leukocytes (Fig. 3a, b). We also measured the maximal respiration along with coupling efficiency of MSCs in the presence as well as absence of leukocytes. Our data indicate that there was no significant difference observed in any of these parameters at different passages (Fig. 3c, d).

### Oxidized phosphatidylcholine (ox-PCs) levels change in MSCs without affecting immunoprivilege

**Fig. 4:**
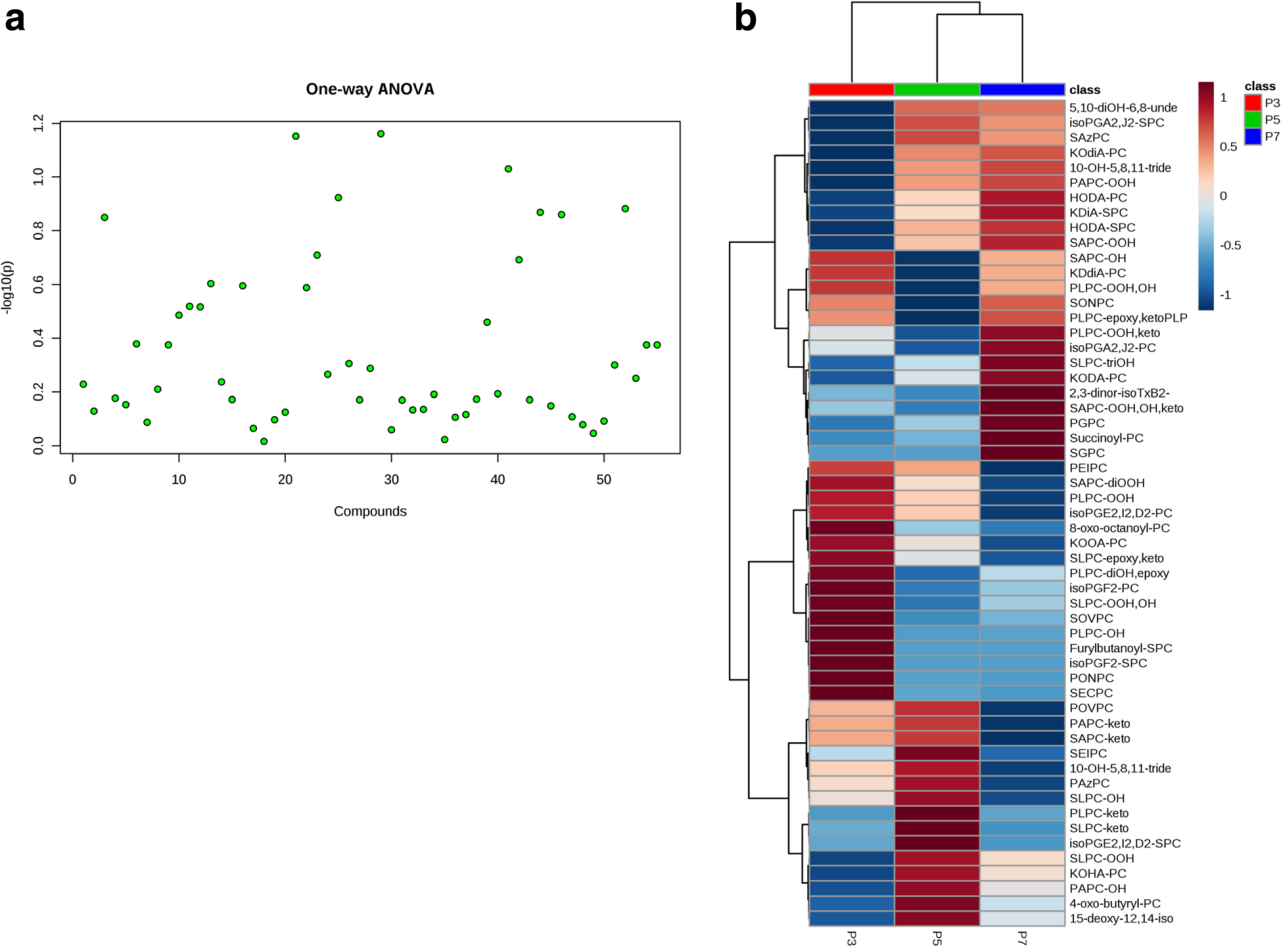
Oxylipidome profile of MSCs by LC/MS was carried out at passages 3, 5, and 7. **a** One-way analysis of variance (ANOVA) plot with a *p* value threshold of 0.05. **b** Clustered heatmap (distance measure using euclidean, and clustering algorithm using ward) showing the intensity of 55 ox-PC compounds. Each row represents data for a specific ox-PC compound and each column represents an individual passage (P3, P5, and P7). All values are log-normalized values of detected abundance for each ox-PC compound. The colors changing from high (red) to low (blue) correspond to the different intensity level of ox-PCs (*n* = 3)

The cellular ox-PCs have been recognized as important mediators of immune signaling. To assess changes in the total cell oxylipidome at different passages and their effect on immunoprivilege of MSCs, the cells at P3, P5, and P7 were subjected to LC/MS analysis. Our data demonstrate that, overall, there were no significant features identified between the passages (Fig. 4a, b). However, some of the ox-PCs which are already reported (in other cell types) to play a significant role in immune cell suppression and were found to be altered with an increase in passage number in the current study are SOVPC, KDdiA SPC, PAPC-OOH, SAPC-keto, and SECPC (Fig. 4b).

To explore whether changes in the levels of ox-PCs have any effect on the immunological behavior of MSCs, we added E06 antibody in the coculture experiments and assessed leukocyte-mediated cytotoxicity and apoptosis in MSCs. The E06 antibody is responsible for blocking cell surface ox-PCs. Our data demonstrate that the presence of the antibody did not have any significant effect on the number of live/dead MSCs and apoptosis after coculture with leukocytes (Fig. 1b, c). Furthermore, the presence of E06 antibody did not cause any difference in cellular bioenergetics. We did not see any significant changes in the basal respiration rate, spare respiratory capacity, coupling efficiency, or maximal respiration rate before and after the addition of the E06 blocking antibody (Fig. 3a–d). Hence, alterations in individual cell surface ox-PCs from P3 to P7 do not affect the immunological properties or cellular bioenergetics of MSCs.

### The stem cell proteome is largely unchanged over different passages

**Fig. 5:**
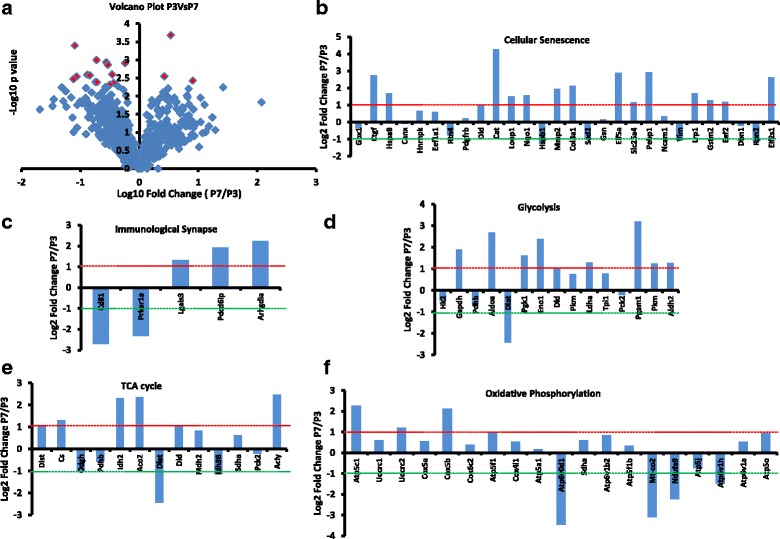
Whole-cell proteome analysis of MSCs at passages 3 and 7 was performed using the LC/MS proteomic platform to determine the changes in different proteins with the increase in passage number. **a** Volcano plot of all the proteins shows no significant changes in all but 18 (shown in red color) proteins (*p* < 0.05). **b**–**f** The values of proteins involved in different pathways including cellular senescence (**b**), immunological synapse (**c**), glycolysis (**d**), tricarboxylic acid (TCA) cycle (**e**), and oxidative phosphorylation (**f**). Log2 fold change ratios of protein values at P7 versus P3 were calculated. The protein values between −1 and + 1 were considered to be normal and not changing significantly. Values higher than +1 indicate significant upregulation (at P7 compared to P3) of the protein and values lower than −1 indicate significant downregulation of the protein levels (*n* = 3)

We performed whole-cell proteomic analysis at different passages using LC/MS to study changes in the levels of intracellular proteins related to cellular senescence, immunogenicity, and bioenergetics. In total, over 800 proteins were screened (Fig. 5a). Our proteomic data recorded some changes in the levels of proteins associated with cellular senescence and aging (Fig. 5b) and immunological synapse (Fig. 5c). Furthermore, some of the proteins that have been reported to play a role in cellular respiration pathways including glycolysis and oxidative phosphorylation as well as tricarboxylic acid (TCA) cycle showed changes with the increase in passage number (Fig. 5d–f). The proteomic analyses for mitochondrial pathways also indicated changes in some proteins in P7 versus P3 (Additional file 2: Figure S2). However, overall, the extent of change recorded in intracellular proteins was not significantly different among P3 and P7, and the changes recorded were not able to affect immunological behavior of MSCs.

## Discussion

In various animal models of degenerative diseases, transplantation of bone marrow-derived allogeneic MSCs has triggered regenerative processes. Based on the encouraging outcome of animal-based studies, several clinical trials have tested the efficacy of allogeneic MSCs. Although the outcome of allogeneic cell-based clinical trials has been encouraging, it is not as effective as the outcome of preclinical studies. Some of the recent studies reported that allogeneic MSCs after transplantation were immunogenic and were rejected by the host immune system. Therefore, understanding the mechanisms of the switch in the immunological behavior of MSCs from immunoprivileged to the immunogenic state would help in preserving the benefits of allogeneic MSCs. The current study is the first to evaluate the possible role of cellular bioenergetics and cell surface ox-PCs in combination with intracellular proteomic analysis in regulating the immunoprivilege of MSCs over different passages. The notion that an increase in passage number in cell culture may affect cellular physiology, morphology, and particularly cell surface molecules is being actively debated. It has been suggested that senescence of MSCs starts as soon as their culturing is initiated. Furthermore, the cells start losing the ability to differentiate, doubling capacity, and telomere length as the passage number increases in cell culture [17]. The outcomes of the majority of MSC-based animal studies and clinical trials have suggested that allogeneic MSCs after transplantation were immunoprivileged and there was no immune response detected in the recipient system against transplanted cells [18, 19]. However, some recent studies revealed that antidonor alloantibodies were detected against transplanted MSCs [3, 20] and cells were rejected by the recipient immune system. One of the important variables in these studies was that cells employed for these studies were from different passages. Therefore, in this study we investigated the effect of an increase in passage number in cell culture on the immunological behavior of MSCs. The most commonly used passages for cell therapy in concluded or ongoing studies include passages 3–7. Therefore, in the current study, we included P3, P5, and P7 for evaluation. Our investigations were focused on hitherto untested parameters of cell surface ox-PCs, cellular bioenergetics, global, and proteomic assessment.

Previous studies have reported an association of cellular senescence with alterations in the immunological behavior of MSCs [21–23]. Senescent cells are reported to attract immune cells; for instance, fibroblasts undergoing senescence secrete various cytokines as well as chemokines which lead to activation of lymphocytes and macrophages [23]. At the organ level, it is reported that kidneys from old donors are more prone to rejection by the host immune system compared with those from young donors [24]. Additionally, in the case of bone marrow-derived MSCs, senescence is associated with a reduced differentiation and proliferation potential [25]. Furthermore, radiation-induced senescence in MSCs is associated with abrogation of the immunomodulatory properties and impaired therapeutic potential in vivo in a mouse model of sepsis [26]. However, the effect of an increase in passage number on immunoprivilege of MSCs has not been investigated thoroughly. Therefore, to investigate this, in the current study we analyzed cellular changes in immunogenicity at different passages. We found that an increase in passage number from P3 to P7 did not have any significant effect on immunoprivilege of MSCs since leukocyte-mediated cytotoxicity and cell death in MSCs did not change between the different passages. Furthermore, there was no significant difference observed in the MSC-mediated suppression of leukocyte proliferation with different passages of MSCs.

Several studies in the literature have reported the role of the cellular bioenergetics profile in the regulation of immune response. The mode of intracellular respiration plays a key role in influencing the presence or absence of T-cell activation signals and thus regulating immunoprivilege of a cell [27, 28]. The choice of fuel (glucose or fatty acids) used for mitochondrial metabolism regulates the interaction of the cell with the immune system [29, 30]. In dendritic cells, there is a switch in the mode of metabolism from oxidative phosphorylation to glycolysis that triggers their activation [31]. In another study, the effect of blocking mitochondrial respiration was reported to reduce the binding of TNF-α to the cells, indicating that mitochondrial respiration might be an important mediator of the immune responses controlled by TNF-α [32]. To assess the effects of an increase in passage number on the cellular respiratory profile in MSCs and its association with the immunoprivilege of MSCs, we performed Seahorse XF24 analysis at different passages. Our data demonstrate that within clinically relevant passages an increase in passage number in cell culture from P3 to P7 did not affect basal respiration rate, spare respiratory capacity, maximal respiration, or coupling efficiency in MSCs before and after coculture with leukocytes.

Another major modulator for the immunogenicity of cells is the expression of cell surface ox-PCs. Several studies have described the role of oxidized phosphatidylcholines in modulating the immunological behavior of immune cells. Oxidation of phospholipids under various conditions such as inflammation, apoptosis, and senescence leads to the generation of proinflammatory damage-associated molecular products (DAMPs) that leads to recognition of the cells expressing these epitopes by the innate immune system [33]. During atherosclerosis, ox-PCs including phosphatidylcholines served as signals for uptake of the cells expressing the phospholipids [34]. However, antagonistic to their role in immune cell activation, oxidized phospholipids are also reported to be involved in preventing the activation of T lymphocytes [35]. Adding to their immunosuppressive role, oxidized phospholipids are also known to prevent the activation of dendritic cells by Toll-like receptor (TLR)3- and TLR4-mediated pathways [36]. These lipids can be both the mediators and the result of cellular apoptosis in different cell types [37]. In various models, the cells expressing the oxidized phospholipids are recognized by macrophages for apoptosis [38]. Interestingly, the macrophages might themselves undergo apoptosis due to activation of the TLR pathway by oxidized phospholipids [39]. Therefore, ox-PCs play an important role in mediating the cellular response to the immune system. To assess the effect of oxidized phospholipids on the immunoprivilege of MSCs in different passages, we performed LC/MS lipidomic analysis. Our data revealed that some of the ox-PCs were changing with the increase in passage number. However, alterations in ox-PCs did not affect immunoprivilege of MSCs. To determine if the ox-PCs were acting in tandem with downstream intracellular protein alterations, we performed proteome analysis using the LC/MS proteomic platform to screen more than 800 proteins. The overall trend recorded in intracellular proteins did not change in MSCs with an increase in passage number. However, we found changes in some proteins involved in cellular senescence and metabolism, but these changes were not able to affect the immunological behavior of MSCs.

## Conclusion

Our study suggests that an increase in passage number from P3 to P7 does not affect immunoprivilege of MSCs. However, more studies are needed to delineate the mechanisms of switch in the immunological behavior of MSCs after transplantation.

## Additional files

Additional file 1: **Figure S1.** Differentiation of MSCs into osteocytes, adipocytes, and chondrocytes. MSCs were induced to differentiate toward osteocytes (a), adipocytes (b), and chondrocytes (c). MSCs (undifferentiated cells, control group) and differentiated MSCs (D-MSC) were stained for osteocalcin and Alizarin Red (osteocyte lineage), FABP4 and Oil Red-O stain (adipocyte lineage), and aggrecan (chondrocyte lineage). The images were taken using Cytation5 (BioTek Instruments) (20× magnification). (*n* = 6) (PPTX 2648 kb)

Additional file 2: **Figure S2.** Heat map showing the highly upregulated or downregulated proteins that are known to be associated with mitochondrial respiration and immune pathways at P3 versus P7. (PPTX 5354 kb)
