# Supplementary material for: Early passaging of mesenchymal stem cells does not instigate significant modifications in their immunological behavior
Source: Stem Cell Res Ther. 2018 May 2;9:121. doi: 10.1186/s13287-018-0867-4 (PMC5930635; doi:10.1186/s13287-018-0867-4)
Supplement: Supplementary file 1 — Figure S1. Differentiation of MSCs into osteocytes, adipocytes, and chondrocytes. MSCs were induced to differentiate toward osteocytes (a), adipocytes (b), and chondrocytes (c). MSCs (undifferentiated cells, control group) and differentiated MSCs (D-MSC) were stained for osteocalcin and Alizarin Red (osteocyte lineage), FABP4 and Oil Red-O stain (adipocyte lineage), and aggrecan (chondrocyte lineage). The images were taken using Cytation5 (BioTek Instruments) (20× magnification). (n = 6) (PPTX 2648 kb) [file 13287_2018_867_MOESM1_ESM.pptx]

## Slide 1
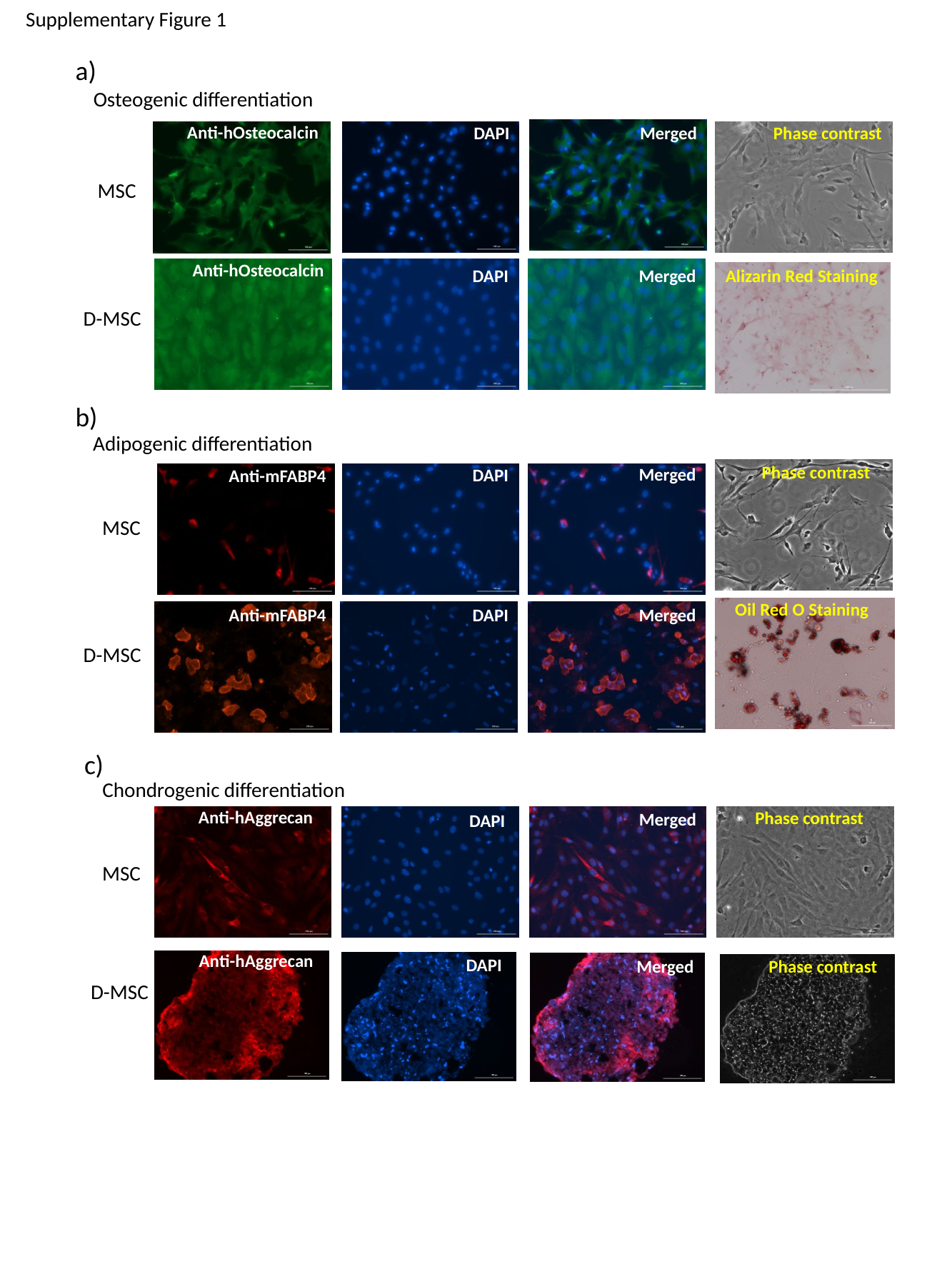

Supplementary Figure 1
a)
Osteogenic differentiation
Anti-hOsteocalcin
Merged
Phase contrast
DAPI
MSC
Anti-hOsteocalcin
DAPI
Merged
Alizarin Red Staining
D-MSC
b)
Adipogenic differentiation
Phase contrast
Merged
DAPI
Anti-mFABP4
MSC
Oil Red O Staining
Anti-mFABP4
DAPI
Merged
D-MSC
c)
Chondrogenic differentiation
Anti-hAggrecan
Phase contrast
Merged
DAPI
MSC
Anti-hAggrecan
DAPI
Merged
Phase contrast
D-MSC
