# Supplementary figures and images for: Early passaging of mesenchymal stem cells does not instigate significant modifications in their immunological behavior
Source: Stem Cell Res Ther. 2018 May 2;9:121. doi: 10.1186/s13287-018-0867-4 (PMC5930635; doi:10.1186/s13287-018-0867-4)

## Slide 1
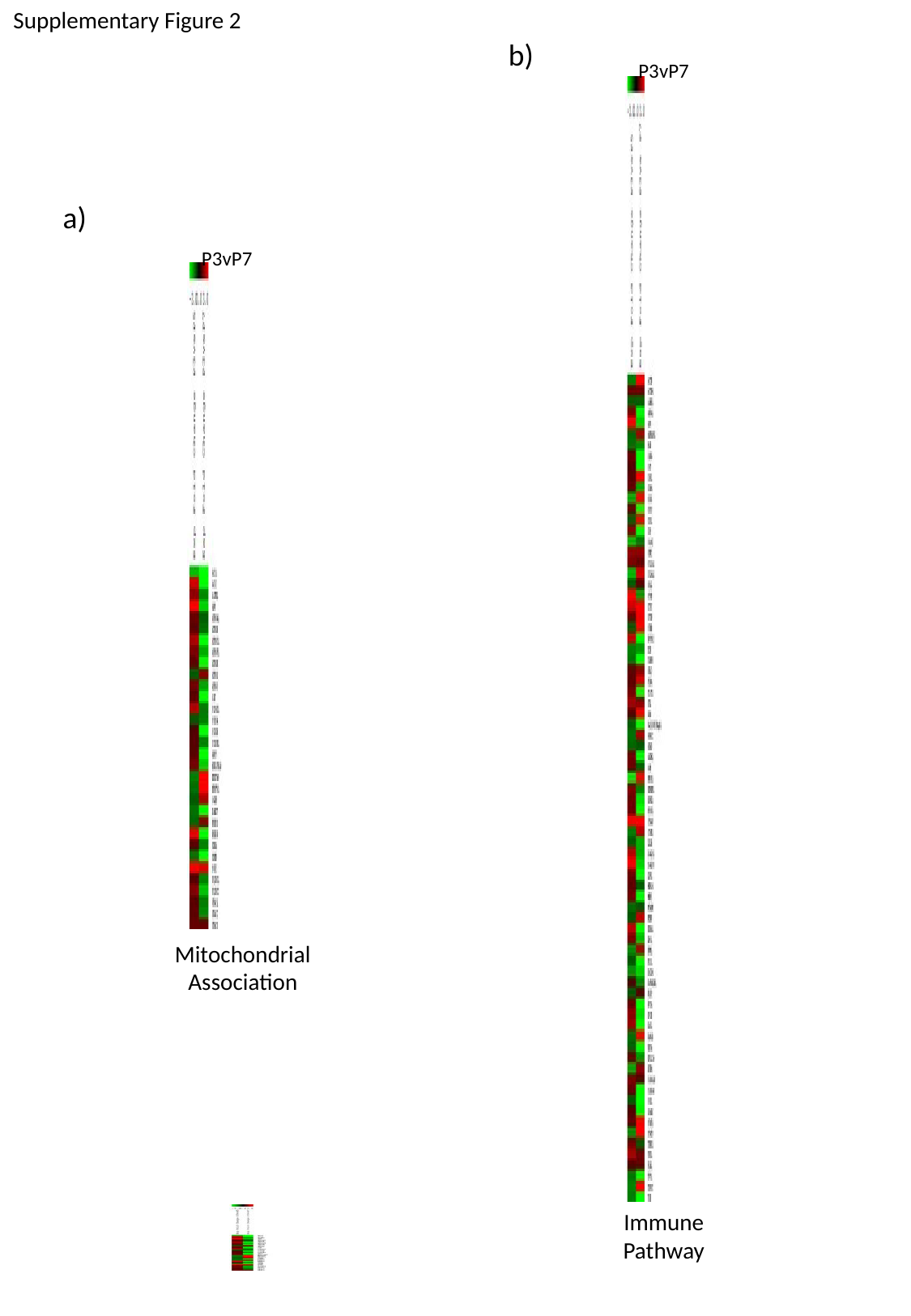

Supplementary Figure 2
b)
P3vP7
a)
P3vP7
Mitochondrial Association
Immune Pathway

Supplement: Supplementary file 2 — Figure S2. Heat map showing the highly upregulated or downregulated proteins that are known to be associated with mitochondrial respiration and immune pathways at P3 versus P7. (PPTX 5354 kb) [file 13287_2018_867_MOESM2_ESM.pptx]
